# Comment: The Balloon-Assisted Double-Kissing T-Stenting Technique: Concept, In Vitro Model, and Case Examples

**DOI:** 10.1016/j.jscai.2024.102197

**Published:** 2024-07-22

**Authors:** Giuseppe Marchese, Ervis Hiso, Giulio Rodinò, Gianluca Rigatelli, Marco Zuin

**Affiliations:** a Interventional Cardiology Unit, Division of Cardiology, Madre Teresa di Calcutta Hospital, Ospedali Riuniti Padova Sud, Monselice, Italy; b Department of Cardiology, Ferrara University Medical School, Ferrara, Italy

We read with interest the article by Rinfret et al^1^ about a novel technique called the balloon-assisted double-kissing T-stenting technique published in *JSCAI*; however, the article raises some issues that are worthy of being discussed. First of all, the technique itself appears to be, for the most part, the same as the nano-inverted T technique published years ago^2^ by our group as a modification of the nano-crush technique^3^ and cited by the European Bifurcation Club consensus.^4^ That technique was conceived to minimize crushing of the side branch (SB) stent protrusion into the main vessel (MV) in order to offer a more physiologic profile compared with other double stenting techniques such as culotte stenting and double-kissing crush stenting^5^ as assessed by bench studies and computational fluid dynamics. With our technique, the SB ostium has proven to be covered by both the SB stent itself and by the MV stent struts projecting into the SB. The nano-inverted T technique has been extensively applied to a wide range of bifurcation lesions, in particular the left main, showing very promising long-term outcomes.^2^ The technique outlined by Rinfret et al^1^ proposes a similar approach with the difference being that the SB stent implantation is aided by the MV inflated balloon, as we usually do with the old T-stenting balloon technique. Unfortunately, using the T-stenting balloon technique causes the ostium of the SB to often be missed because the inflation of the MV balloon pushes the SB stent slightly away from the anatomic SB ostium, accounting for the poor results of T-stenting in real practice. Finally, the bench study associated with the clinical application of the Rinfret technique actually included very simple bifurcation angles, which do not appear challenging enough to prove that the technique effectively covered the ostium.

## Funding sources

This work was not supported by funding agencies in the public, commercial, or not-for-profit sectors.

## Declaration of competing interest

The authors declared no potential conflicts of interest with respect to the research, authorship, and/or publication of this article.
